# Increased initial task difficulty drives social foragers to develop sub-optimal conformity instead of adaptive diversity

**DOI:** 10.1098/rsos.230715

**Published:** 2023-07-05

**Authors:** Đorđe Marković, Na'ama Aljadeff, Lucy M. Aplin, Arnon Lotem

**Affiliations:** ^1^ School of Zoology, Faculty of Life Sciences, Tel Aviv University, Tel-Aviv, Israel; ^2^ Department of Evolutionary Biology & Environmental Studies, University of Zurich, Zurich, Switzerland; ^3^ Research School of Biology, Australian National University, Canberra, Australia

**Keywords:** social learning, conformity, social foraging, cognition

## Abstract

The extent to which animal societies exhibit social conformity as opposed to behavioural diversity is commonly attributed to adaptive learning strategies. Less attention is given to the possibility that the relative difficulty of learning a task socially as opposed to individually can be critical for social learning dynamics. Here we show that by raising initial task difficulty, house sparrows previously shown to exhibit adaptive social diversity become predominantly conformists. The task we used required opening feeding well covers (easier to learn socially) and to choose the covers with the rewarding cues (easy to learn individually). We replicated a previous study where sparrows exhibited adaptive diversity, but did not pre-train the naive sparrows to open covers, making the task initially more difficult. In sharp contrast to the previous study results, most sparrows continued to conform to the demonstrated cue even after experiencing greater success with the alternative rewarding cue for which competition was less intense. Thus, our study shows that a task's cognitive demands, such as the initial dependency on social demonstration, can change the entire learning dynamics, causing social animals to exhibit sub-optimal social conformity rather than adaptive diversity under otherwise identical conditions.

## 1. Introduction

The interplay between social foraging, social learning, and the development of animal culture has been the focus of growing interest [1–7]. During social foraging, animals may learn through observation or interaction with others, which is commonly referred to as social learning [8]. Using social learning may be adaptive as it can improve foraging success, facilitate the acquisition of new skills or help to avoid predators (reviewed by [9]). It may also result in the formation of culture if it enables the accumulation, or spread, of shared group behaviours [10,11]. Yet, the expression of social learning is quite variable, partly because animals apply a variety of social learning strategies instructing them when, what, and whom they should, or should not copy [12,13]. These strategies have presumably evolved (or developed) because learning from others may not always be better (or more economical) than relying on self-experience, and even when it is, it makes sense to learn only from individuals who are better informed, more skilled, or with relevant experience.

The existence of different social learning strategies, for which evidence is accumulating ([13–15], but see [16]) implies that such strategies can determine how information is transmitted and shared among group members, and how likely, therefore, it is that a uniform culture would emerge and be maintained. For example, offspring who learn from their parents generate mainly vertical transmission of information in the population (e.g. [17]), while young individuals who learn from any adult generate oblique transmission (e.g. [18]), which may accelerate information sharing. On the other hand, a tendency to learn only from similar individuals (assortative social learning) may sub-divide information sharing and result in cultural diversity within populations [19,20]. In the simple case where a new behaviour is rewarding and easy to copy, a rapid spread of this behaviour is expected, forming a behaviourally uniform group (or population) with a distinct culture of its own.

However, when two or more alternative behavioural variants appear in the population, the formation and maintenance of distinct singular cultures have been suggested to be more probable if individuals follow a conformist learning strategy [21,22], that is, a social learning strategy in which individuals acquire behaviour (or change existing behaviour) with a positive frequency-dependent learning bias, preferentially copying the most common behaviour or the majority of individuals [23,24]. Conformity has been studied in the past mainly in relation to human societies [24], but its occurrence in animals has been recently demonstrated in primates [25,26], fishes [27], and birds [28].

Conformity has been used to describe several related phenomena [29,30]. In the broad sense that will be used in this paper (and as commonly used in spoken language), conformity, or the tendency to conform, refers simply to ‘copying the most common behaviour’ regardless of the exact mechanism leading to it. Narrower definitions of conformity have included a tendency to copy the majority (or the common behaviour) with a probability that is greater than its current proportion [31–33], or a tendency to change existing behaviour to match a majority [24,25,29]. These extra conditions are clearly of interest, but require quantification of the learning process in a way that is not always possible. In all definitions, however, conformity leads to behaviourally uniform groups, helps to maintain distinct cultures, and may be viewed as adaptive under the assumption that the commonly exhibited behaviour is more likely to be adaptive than the rarely exhibited one. Importantly, this last assumption may not always be correct.

It should be clear that conformity cannot be adaptive if the most common behaviour in the population is no longer adaptive. This has been suggested to occur when individuals compete over limited resources, causing the adaptive value of a demonstrated behaviour to decline in a negative frequency-dependent fashion; the more common the behaviour, the greater the competition for the resource, which lowers the adaptive value of this common behaviour, as well as the adaptive value of conformity [34,35]. Moreover, it has also been suggested that in a diverse habitat, where food patches offer more than one type of food, conformity that creates groups of uniform specialists may be inefficient, whereas negative frequency-dependent learning that creates groups of complementary specialists (i.e. a skill pool) is expected to increase foraging success for both individuals and groups [34]. These theoretical predictions have been tested recently by Aljadeff *et al*. [36] in house sparrows (Passer domesticus). They found that under competitive conditions of food depletion, naive sparrows introduced into groups of foraging specialists did not conform to the behaviour of the specialists, but rather learned to use the alternative food-related cues for which competition was less intense. They also showed that this learning dynamic created groups of complementary specialists that enjoyed greater foraging success than sparrows in uniform groups [36]. Thus, it seems that when there are multiple foraging options, and the rarely used one is advantageous due to reduced competition, social conformity may be replaced by adaptive diversity.

Yet, as previously hypothesized [36], another critical factor that may determine the extent to which social conformity or competitive diversity are expressed in the population is the relative ease of learning the foraging tasks socially as opposed to individually. It is quite possible, for example, that the strong conformity exhibited by great tits (*Parus major*) when learning to open a puzzle box [28] as opposed to the diversity exhibited by house sparrows in the abovementioned study [36] was not only due to food depletion in the latter case, but also to the different cognitive demands imposed by the different tasks. Learning to open the puzzle box in the great tits study was clearly difficult without social demonstration [28] while the sparrows that exhibited diversity in the abovementioned study were pre-trained to open the feeding wells and only needed to learn the rewarding cues [36]. Thus, the sparrows could easily explore alternative options right from the start (which they did – see [36]), while the great tits were much more likely to apply the demonstrated solution, and since it was always rewarding (the puzzle box feeder was never depleted) they continued to conform. In the absence of food depletion conformity was indeed adaptive. Yet, even if food is occasionally depleted, adaptive diversity may not develop unless it is possible for the birds to learn and to experience success in the alternative task. For example, in a previous study on blue tits (*Cyanistes caeruleus*), almost all individuals used only the demonstrated solution despite its depletion, leaving the alternative food resources untouched [37]. This effect of task-related cognitive demands may not be restricted to the case where asocial learning is impossible. It is perhaps sufficient that initial difficulties (and failures) when trying to learn asocially shift the learner's attention to other individuals, thereby increasing the likelihood of social learning and the weight given to social information (see also [38]).

Here we experimentally tested this possibility by raising initial task difficulty under otherwise identical conditions to those previously used to demonstrate adaptive diversity in our studied population (by Aljadeff *et al*. [36]). Our experimental set-up was based on foraging grids of feeding wells that allow house sparrows to compete for a limited amount of food. The task we used required opening feeding well covers (easier to learn socially than asocially [37,39]) and to choose the covers with the rewarding cues (easy to learn individually [36,40]). In the previous study, the naïve birds that were introduced into groups of skilled demonstrators were pre-trained to open unmarked covers of feeding wells through a gradual shaping process (see Methods and [36]). Thus, they only needed to learn which of the cues printed on the covers indicate the presence of food [36]. Here, on the other hand, we increased initial task difficulty by omitting the pre-training stage, so that naive sparrows had to learn both the action of cover opening (which is difficult to learn without shaping or social demonstration), as well as the rewarding cues. As in the previous study, we allowed the learners and the demonstrators to jointly forage on a grid containing rewarding and non-rewarding colours and shapes, and we monitored their behaviour. The demonstrators in each group were pre-trained to specialize on either the rewarding colour or the rewarding shape, demonstrating cover opening of only one of the two types. We examined whether in contrast to the previous study results, the naive sparrows in the present study exhibited social conformity rather than diversity, and whether they persisted with sub-optimal conformity even after experiencing greater success with the alternative rewarding cue for which competition is less intense.

## 2. Methods

### 2.1. Study population and general setup

During the fall of 2020, 120 adult house sparrows (*Passer domesticus*; 60 males and 60 females) were caught at Beit Kama's cattle station, south Israel, and brought to the I. Meier Segals Garden for Zoological Research, Tel Aviv University, Israel. House sparrows are abundant and considered agricultural pests in Israel, and are therefore legally unprotected. The experiments were conducted under a permit from Tel-Aviv University Institutional Animal Care and Use Committee (04-19-062).

The sparrows were first housed in four outdoor aviaries (4 × 4 × 3 m each, Length×width×height, 30 sparrows in a cage). They were given food (a mixture of commercial bird feeds, shredded boiled eggs, occasionally insects) and water ad libitum on a daily basis (for more details on our captive colony, see [36,41–43]). All sparrows were individually marked by an aluminium ring and by colouring their tail feathers with an odourless, non-toxic, marking ink (Uni-posca paint marker). After the experiments, the birds were kept in the large aviaries for several weeks (during which their tail colours wore off), and were then released in good shape (based on examination of plumage and body mass) in the area of I. Meier Segals Garden for Zoological Research, Tel Aviv University.

After two weeks of habituation to captivity, small flocks of birds (see details in the following section) were transferred to one of three experimental cages (3.5 × 3 × 3 m each). A foraging grid—a 140 × 140 cm wooden panel containing 144 feeding wells (2.5 cm in diameter, 1 cm depth, and 11.5 cm centre to centre)—was placed in the middle of the floor of each experimental cage and was exposed to the sparrows during the shaping, training, and experimental sessions that will be described below. The birds' activity on the foraging grid was recorded with an HD video camera through a one-way window.

The feeding wells of the foraging grid could be covered with a paper on which colour or shape cues could be printed, and in these cases the birds had to learn to peck through a slit in the covers, and to choose the covers with the right cues in order to find hidden millet seeds (see further details below). To habituate the sparrows to the foraging grid, they were initially fed from standard feeding trays placed on the surface of the foraging grid, and then 3–4 times a day the feeding trays were removed and millet seeds (used as rewarding food in the following stages) were placed in the feeding wells of the foraging grid (that were not yet covered at this stage). To keep the sparrows motivated to forage on the grid throughout the experiments, we deprived them of food during the two hours preceding the first experimental session of each day, and returned the food dishes to the cage only after the fourth experimental session of the day. Thus, from the 2 h preceding the first session and until the end of fourth session (approx. 5 h in total), the only source of food the sparrows could access was the hidden millet seeds on the foraging grid.

### 2.2. Experimental design

#### 2.2.1. General design

The experiments were based on 10 cohorts of 16 individuals (8 female and 8 males in each). Within each cohort, birds were randomly assigned to one of three groups: two groups of six birds that were trained as specialists (specializing in opening covers with a specific cue, see below), and one group of four birds that were kept as naive.

**Figure 1.**
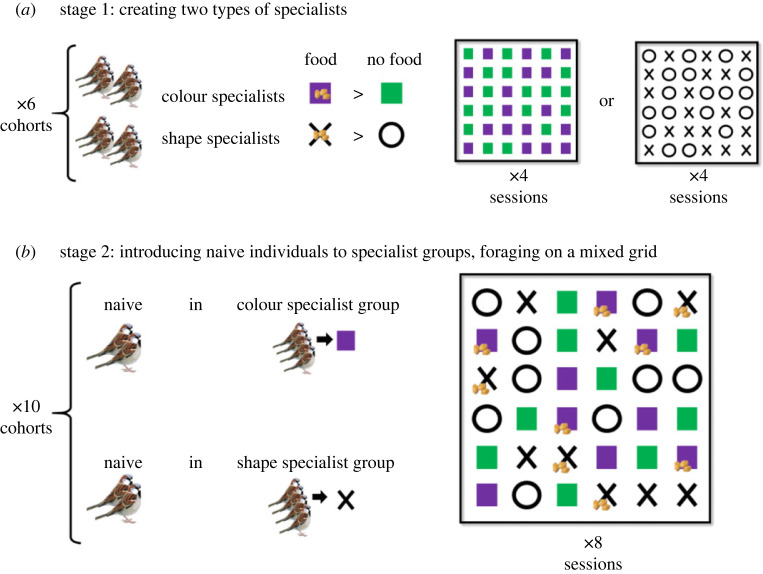
Experimental design. For ease of illustration, foraging grids are illustrated here with only 6 by 6 feeding wells, and with only one colour and one shape as rewarding (purple and X). In practice, each grid contained 12 by 12 feeding wells and the roles of colours and shapes were counterbalanced across cohorts (see Methods). (*a*) Stage 1: sparrow flocks were trained to specialize in either colour or shape, during which the foraging grid contained either two colours or two shapes, one rewarding in half of the cases (7 seeds, *p* = 0.5) and the other never rewarding (no seeds). (*b*) Stage 2: two naive individuals were introduced into two types of specialist groups (colour and shape) and foraged with them on a grid containing both rewarding and non-rewarding colours and shapes during eight successive sessions. To avoid pseudo-replications and specific spatial effects we used different spatial distributions of cues for each foraging session. All stages were repeated on 10 different cohorts (see Methods for more details).

As in Aljadeff *et al.* [36], the birds in the specialists groups were first trained to become specialists, so they could serve as the demonstrators for those in the naive group, when later tested together during the experimental stage (see schematic description of the experimental design in figure 1). To that end, the birds in the specialists groups went through a pre-experimental stage that included shaping and training. During shaping the specialists learned to open the paper covers, and during training they learned which cue (one of two colours or one of two shapes) indicated the presence of food. The shaping and the training procedures of the specialists during the pre-experimental stage are described in detail in the following sub-section. During the same pre-experimental stage, the naive birds did not go through the shaping and training process and were merely habituated to forage on the foraging grid when the feeding wells were exposed (i.e. not covered). Importantly, this procedure differed from that used by Aljadeff *et al.* [36] where the birds in the naive groups did go through a shaping process with unmarked (white) covers. As a result, by the experimental stage, the naive birds in Aljadeff *et al*. were naive only in respect to the relevant food-related cues which they could then learn socially from the specialists, or asocially from their own experience. By contrast, the naive birds in the present study faced a more difficult task at the onset of the experimental stage: they had to learn first the action of opening covers, and simultaneously, or subsequently, the correct cues that indicate the presence of food.

For the experimental stage we created mixed groups of two naive individuals and four specialists. The specialists were pre-trained to demonstrate either the correct colour (colour specialists) or the correct shape (shape specialists) but the foraging grid included both rewarding and unrewarding colours and shapes (figure 1). Thus, during the experimental stage we could examine whether the naive learned socially from the four specialists and competed with them over the same type of feeding wells (e.g. rewarding shapes in shape specialist groups), or rather they learned from their own experience to prefer the alternative rewarding cue (e.g. preferring the rewarding colour in shape specialist groups) as in Aljadeff *et al*. [36]. We introduced two naive individuals to each specialist group rather than a single one, to secure sufficient behavioural data from at least one of the two.

#### 2.2.2. Stage 1: pre-experimental shaping and training of specialists

We first used a shaping process which allowed the specialists in each cohort (two groups of six birds) to learn to open the paper covers. To that end, the feeding wells of the foraging grid, that provided 2–3 millet seeds each, were covered with a slotted white paper in a manner presenting increasing degrees of difficulty—from widely open slits to completely closed ones. The shaping took four sessions after which most birds learned to open the covers where a session was a single round in which the foraging grid with its 144 feeding wells is provided to the birds.

After the shaping, the specialists went through four training sessions during which they learned to prefer one of two food-related cues (colour or shape). Two different colours (purple and green) and shapes (X and O) were printed on the covers. One of the two specialists groups in a cohort learned to prefer one of the colours (purple over green or vice versa; counterbalanced across cohorts) and the other group learned to prefer one of the two shapes (X over O or vice versa; counterbalanced across cohorts; figure 1*a*). For both the training and the experimental stage, half of the wells with the rewarding cue contained 7 millet seeds (0.5 reward probability), whereas the wells with the non-rewarding cue were always without any seeds. We used a partial reward regime for the rewarding cue (7 seeds with probability of 0.5) in order to increase the tolerance of the specialists to occasional failure, which is both realistic in nature and expected to occur during the experiment as a result of food depletion. Indeed, it resulted in strong and persistent preference for one cue by the specialists both in Aljadeff *et al*. [36] and in the present study (see Results).

The four training sessions lasted up to 5 min each or until approximately 75% of the wells with the rewarding cues were visited. At the end of the training sessions, the four of the six specialists with the highest rewarding to non-rewarding peck ratio were selected for the following experimental stage as potential demonstrators, while the remaining two were returned to the colony and later released. To save training time and numbers of trained specialists, we used the same two quartets of specialists in cohorts 3 and 4, 5 and 6, 7 and 8, and 9 and 10. Thus, overall we trained 72 specialists of which we used 48 in the 10 cohorts of the experimental stage (8 in cohort 1, 8 in 2, 8 in 3 and 4, 8 in 5 and 6, 8 in 7 and 8 and 8 in 9 and 10). There was no such overlapping use of naive birds: in each of the 10 cohorts we used different quartets of naive birds (*n* = 40).

#### 2.2.3. Stage 2: the experimental stage

For the experimental stage we created groups of four specialists and two naive birds that were housed together at the same aviary and foraged together on the grid. In each cohort we created two such groups: one with colour specialists and one with shape specialists. For both groups, the foraging grid contained all four food-related cues (green, purple, O and X, 36 of each, 144 wells in total) but only one colour and one shape were rewarding (7 millet seeds with a probability of 0.5, see above) while the others were not (no seeds in all cases). Importantly, in each group, the colour or the shape that the specialists of this group learned to prefer during the pre-experimental stage was also used as the rewarding cue during the experimental stage. This allowed the specialists to continuously demonstrate their preference for this cue to the naive birds. The other rewarding cue was chosen at random but was counter balanced across groups. It provided an alternative rewarding cue for the naive birds to learn without social demonstration.

The experimental stage involved eight consecutive sessions and lasted for two days (four sessions per day, 35–45 min apart, starting at 9 : 00 or 13 : 00, randomized across colour and shape groups). Before each session, feeding wells were assigned colour and shape lids randomly and refilled with seeds as prescribed (figure 1*b*). A session began by exposing the foraging grid and lasted 4 min, with additional 1.5 min given if less than approximately 50% of the rewarding wells were consumed. The experimental stage in our study was twice as long as in Aljadeff *et al*. [36] to allow the naive birds to a) learn to open the covers (which they were already pre-trained to do in Aljadeff *et al*.) and b) have enough time to explore and shift to the alternative rewarding cue for which competition was less intense, even if they initially learn to open the cover with the cue that was demonstrated to them by the specialists. Thus, the experimental design allowed us to test whether increasing initial task difficulty (no pre-training of cover opening) changed learning dynamics and skills distribution in sparrows groups in comparison to those tested under otherwise identical conditions by Aljadeff *et al*. [36].

### 2.3. Behavioural and data analysis

All training and test sessions were video recorded to enable a step-by-step analysis of foraging decisions. We followed each bird separately from the time it landed on the grid until it left. We included in our analysis only birds that opened at least six feeding wells (of any of the cues) during the entire experimental stage. This criterion was chosen *a priori* based on previous studies [36,43] and resulted in 18 out of the 40 naive birds that foraged on the grid to be removed from the analysis, leaving *n* = 22 naive birds. We defined a foraging step as a visit to an intact well that included at least one peck by the focal bird. Intact wells were those that had not been already pecked at by any of the sparrows (including the focal bird) and were therefore rewarding if initially containing seeds. In the analysis, we discriminated between intact and open wells and used only pecks at an intact well to score colour or shape preference. This is because pecking at open wells can also possibly be triggered by an inclination to indirect scrounging.

To control for momentary changes in the relative availability of intact wells of different types (e.g. rewarding cues preferred by specialists are taken faster) we used our video recordings to track the relative availability of different intact cues prior to each foraging decision made by the focal bird. Thus, we obtained the number of available intact wells of each of the four cues, for each foraging step, and could then compute the expected number of choices for each cue at random and score unbiased preference according to the ratio between ‘observed choices’ (the actual number of choices in each cue) and the expected one (see detailed description of this method and a numerical example in electronic supplementary material, table S1, following Aljadeff *et al*. [36]). Unless otherwise specified, we used this observed-to-expected ratio as an unbiased preference score throughout our analysis. Note that we did not test the statistical significance of each individual's deviation from choices expected at random, as this would result in considerable multiple testing. We merely used these preference scores as unbiased data points for the analysis (similar to using the number or the proportion of choices).

Video analysis was carried out using a Python-based software (*Poke-a-bird 0.7*, http://arnonlotem.weebly.com/technical-tools--code.html) developed by Michal Keren.

Statistical analyses were performed in R v.3.5.1 (R Core Team, 2018). We used non-parametric statistics throughout the paper because our data did not meet the requirements for parametric statistics. As explained above, to avoid depletion bias, unless otherwise specified all analyses were based on the observed-to-expected ratio preference score. All statistical tests are two-tailed unless otherwise specified.

## 3. Results

The specialists that were trained in Stage 1 and participated in Stage 2 maintained clear preference for their target colour or shape during the eight sessions of Stage 2 (see the electronic supplementary material, figure S1). This result confirmed that the experimental design had successfully created the intended social environment for the tested naive individuals as in Aljadeff *et al*. [36]. Namely, shape specialists demonstrated cover openings of the rewarding shape while colour specialists demonstrated cover opening of the rewarding colour.

### 3.1. Naive sparrows conformed to the demonstrated cue

**Figure 2.**
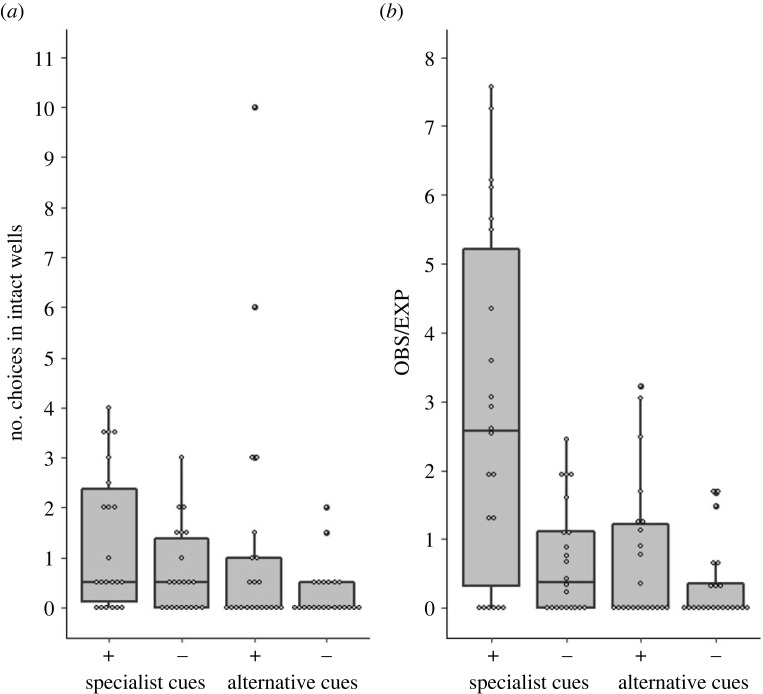
Naive individuals conformed to the demonstrated cue. The distribution of choices by naive sparrows that were introduced into specialist groups (pooled for colour or shape groups) during the last two foraging session of the experimental stage 2 (pooled for sessions 7 and 8). Choices are shown as (*a*) number of pecks in intact wells and (*b*) observed-to-expected ratio (see Methods). Choices were classified as: specialist rewarding (+) and non-rewarding (−) cues, and alternative rewarding (+) and non-rewarding (−) cues (each class contained both shape and colour in a balanced design). Specialist rewarding is the demonstrated cue. Data are represented as median, 25%, and 75% quantiles, and data points (*n* = 22 naive sparrows).

By the end of the experimental stage (during sessions 7 and 8), the naive individuals had shown a clear preference for the demonstrated rewarding cue (figure 2). This was already indicated by the raw data, which was not yet corrected for the lower availability of wells of the demonstrated cue (figure 2*a*), and was highly significant for the corrected data (figure 2*b*; a higher observed-to-expected ratio for the demonstrated than for the alternative rewarding cue: Wilcoxon Signed Rank Test, *n* = 22, *p* = 0.0056).

**Figure 3.**
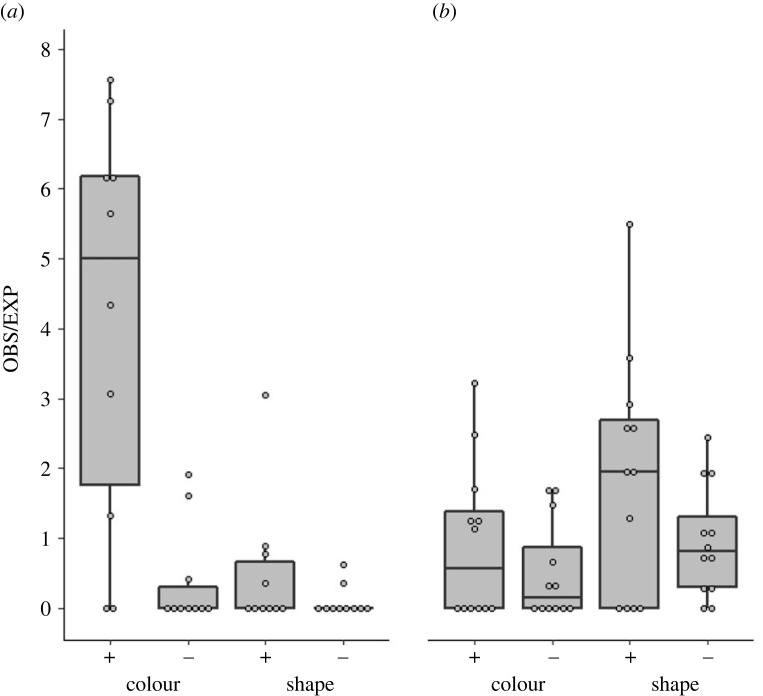
The effect of different types of specialists on naive individuals. The preference scores (observed-to-expected ratio) of naive sparrows in colour and shape specialists groups, measured during sessions 7–8 of the experimental stage: (*a*) colour specialist groups (*n* = 10), (*b*) shape specialist groups (*n* = 12). Available choices: rewarding (+) and non-rewarding (−) colour, and rewarding (+) and non-rewarding (−) shape. Data are represented as median, 25%, and 75% quantiles, and data points.

The preference for the demonstrated rewarding cue was indicated in both colour and shape specialists groups, though it was more pronounced in colour specialists groups. Naive birds in colour specialists groups preferred the rewarding colour (figure 3*a*; a higher observed-to-expected ratio for the demonstrated colour than for the rewarding shape: Wilcoxon Signed Rank Test, *n* = 10, *p* = 0.012), while naive birds in shape specialists groups had a tendency to prefer the rewarding shape, but not significantly so (figure 3*b*; Wilcoxon Signed Rank Test, *n* = 12 *p* = 0.16). The stronger preference of the rewarding colour in the coulor specialists group (figure 3*a*) as opposed to the weaker preference of the rewarding shape in the shape specialists group (figure 3*b*) is consistent with previous findings showing that the colour task is easier for the sparrows than the shape task [40].

Because 12 of the 22 naive individuals that were included in our analysis were composed of 6 pairs of naive individuals tested together in the same group of specialists, there was a possibility that their choices were not independent. We therefore ran a more conservative analysis in which we used the mean observed-to-expected ratio scores of each pair, thus reducing the sample size to 16 (i.e. each pair was taken as an individual). The preference for the demonstrated rewarding cue (figure 2*b*) remained significant (Wilcoxon Signed Rank Test, *n* = 16, *p* = 0.013). Similar results are obtained when choosing the first individual in each pair that opened an intact well during the experiment and ignoring the other one (Wilcoxon Signed Rank Test, *n* = 16, *p* = 0.041).

Thus, in sharp contrast to the results shown by Aljadeff *et al*. [36], where naive sparrows had developed a preference for the alternative rewarding cue, by the end of the experimental stage (sessions 7 and 8) our naive sparrows developed a tendency to conform to the demonstrated cue. Finally, the difference between the two studies is also clearly indicated by simply comparing the number of choices in each cue, and is thus independent of our use of the observed-to-expected score method. Comparing figure 2*a* in the present study with figure 2*a* in Aljadef *et al*. [36], the pattern is strikingly different, and the mean proportion of choosing the demonstrated rewarding cue out of the total number of rewarding cues (demonstrated and alternative) was significantly higher in the present study (0.66 versus 0.17, Mann-Whitney *U* test, *U* = 30, *N*_1,2_ = 18,12, *p* = 0.00104).

### 3.2. Naive sparrows failed to learn to prefer the alternative rewarding cue for which competition was less intense

**Figure 4.**
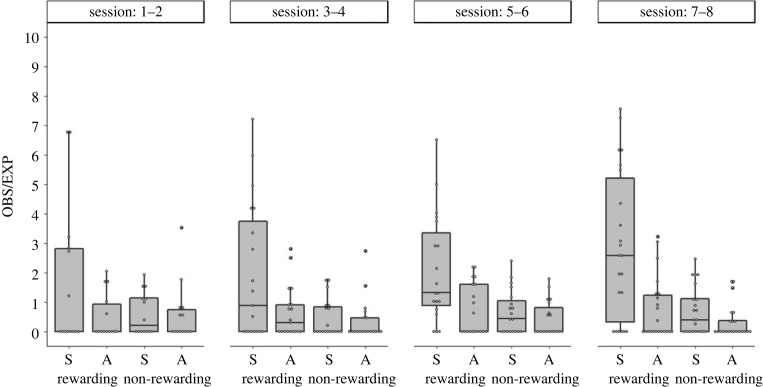
Learning dynamics of naive individuals. The preference scores (observed-to-expected ratio) of naive sparrows that were introduced into specialist groups (i.e. colour or shape) during the eight foraging sessions of the experimental stage (sessions are aggregated in pairs, with *n* = 14, 18, 19, and 22, for sessions 1–2, 3–4, 5–6, and 7–8, respectively). Available choices: Specialists (S) and Alternative (A) rewarding, and Specialists (S) and Alternative (A) non-rewarding. Data are represented as median, 25%, and 75% quantiles, and data points.

To explore the learning dynamics of the naive individuals (that led them to conform by the end of the experiment), we examined their preference scores during the eight sessions of the experiment (figure 4). A preference for the demonstrated rewarding cue is already observed in the first four sessions (sessions 1–4) but it fails to reach statistical significance (partly because of the smaller number of naive birds that learned to open the covers by this stage and the small number of opening performed by each of them). During sessions 5–6, the preference for the demonstrated over the alternative rewarding cue was nearly significant (Wilcoxon Signed Rank Test, *n* = 19, *p* = 0.059), and as mentioned earlier (figure 2 and above), it was clearly significant by the last two sessions of the experiments. Thus, it is possible that most naive sparrows developed a preference for the demonstrated cue as soon as they learned to open the covers but the limited sample sizes during the early sessions does not allow us to verify whether this is really the case.

The preference for the demonstrated cue could have possibly developed even before the first cover opening. This could have happened as a result of delayed scrounging (visiting wells previously opened by the specialists and finding some remaining seeds). This may also explain the greater conformity in colour than in shape (figure 3) because colours are more visible than shapes after cover opening. To examine this possibility we tested the correlation between the number of delayed scrounging visits of a naive bird that occurred before first cover opening, and the preference for the demonstrated cue developed after that. We used three different ways to measure this preference (time to first opening of a demonstrated cue, total number opened, and observed-to-expected preference score). The results (see the electronic supplementary material, figure S2) are inconsistent with the possibility that the preference for the demonstrated cue is due to delayed scrounging. First, delayed scrounging was correlated with slower rather than faster learning (electronic supplementary material, figure S2a), and second, it was inversely related to the number of covers opened, and to the mean observed-to-expected preference score of the demonstrated cue (electronic supplementary material, figures S2b, S2c). Simply put, some naive sparrows were slow to learn to open covers despite extensive experience with delayed scrounging, while others learned to open covers and developed strong preference for the demonstrated cue with no, or very little, delayed scrounging experience (see electronic supplementary material, figure S2). Thus, the effect of delayed scrounging, if it occurred, was marginal relative to the effect of observational learning (see Discussion).

**Figure 5.**
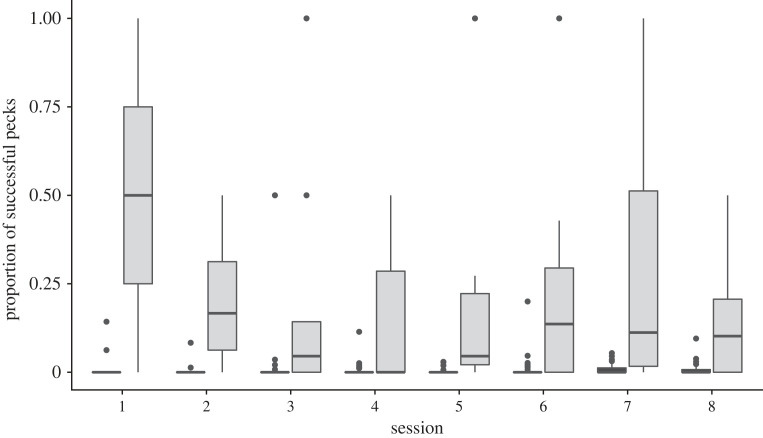
Naive individuals experienced higher success using the alternative rewarding cue. The estimated proportion of successful pecks in wells of the rewarding demonstrated cue (black boxplots) and in wells of the alternative rewarding cue (gray boxplots) by naive individuals during the eight sessions of the experimental stage. The proportion of successful pecks was estimated conservatively as the proportion of pecks in intact rewarding wells (that contained 7 seeds) out of the total number of pecks for that cue (including intact wells of the rewarding cue that did not contain seeds, and open wells that were likely to be empty or partly empty). Data are represented as median, 25%, and 75% quantiles, and data points outside this range. Medians of the alternative rewarding cues were higher than of the demonstrated cues in seven of the eight sessions (binomial test, *p* = 0.0385), and pooling the data for all eight sessions shows that the proportion of successful pecks experienced by naive individuals was higher for the alternative rewarding cue than for the demonstrated cue (Wilcoxon Signed Rank Test, *n* = 22, *p* < 0.0005).

**Figure 6.**
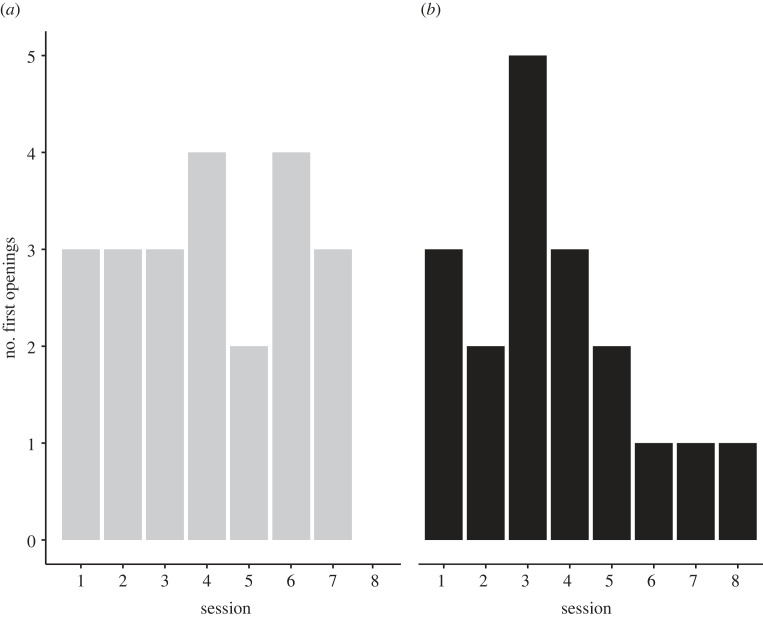
The distribution of first openings of intact rewarding wells by naive individuals during the eight sessions of the experimental stage: (*a*) first openings of wells with the demonstrated rewarding cue (by the 21 naive individuals that opened such wells), (*b*) first openings of wells with the alternative rewarding cue (by the 18 naive individuals that opened such wells).

The results also show that most naive sparrows (18 out of the 22) did open wells of the alternative rewarding cue but their tendency to do so did not increase over time (Friedman test comparing sessions 3–4, 5–6 and 7–8, for birds pecking at alternative rewarding wells in all these sessions, *n* = 9, *χ*^2^ = 1.722, d.f. = 2, *p* = 0.423; figure 4). Estimating the relative success experienced by sparrows when visiting wells of the alternative rewarding cue as opposed to the demonstrated rewarding cue (based on the proportion of intact wells that contained seven seeds out of the total number of pecks for that cue, including open wells that were likely to be empty or partly empty) show that it was clearly higher when visiting wells of the alternative rewarding cue (figure 5 and statistics therein). This result was expected due to the rapid depletion of the demonstrated wells by the specialists and is similar to that found by Aljadeff *et al*. [36]. However, in our study this higher success did not cause the sparrows to shift to the alternative rewarding cue as in Aljadeff *et al*.'s study. The distribution of first openings of demonstrated and alternative rewarding cues by the naive sparrows (figure 6) shows that most sparrows that opened wells of the alternative rewarding cue (13 out of 18 in our sample) did it already at the beginning of the experiment (sessions 1–4), suggesting that they had sufficient time to learn that it yielded a higher success rate.

## 4. Discussion

In this study we examined whether increasing initial task difficulty, in comparison to a previous study, can cause sparrows to exhibit social conformity instead of adaptive diversity. Our results were consistent with this prediction. Naive sparrows introduced into groups of specialized individuals largely conformed to the behaviour demonstrated by the specialists, despite suffering competitive food depletion that makes the alternative rewarding cue more profitable. Moreover, the sparrows persisted with this sub-optimal conformity until the end of the experimental stage that lasted for eight sessions. This was in contrast to a previous study with a lower initial task difficulty, where sparrows exhibited an increasing preference for the alternative rewarding cue within only four experimental sessions [36]. Importantly, the conformity exhibited by the sparrows was not simply due to their inability to open covers of the alternative rewarding cue (as in [37]). On the contrary, the sparrows did manage to open all four types of feeding wells (see figures 4 and 6), and to experience greater success when visiting wells of the alternative rewarding cue (figure 5). Nevertheless, they continued to prefer the demonstrated rewarding cue for which the success rate was lower.

One issue to consider before discussing the results further is the possibility that the contrasting results between the two studies may be related to some other differences, rather than the intended change in initial task difficulty. Although the two studies were conducted at the same place and by the same research group, and carefully followed the same protocol, it is still possible that the sparrows caught four years earlier for the previous study were somehow different, or that another unknown factor affected their behaviour. While it is difficult to rule out this possibility we find it very unlikely that such unknown differences could explain the contrasting results. In both studies, the specialists that were trained in the same way also behaved in the same way, showing no indication for differences in learning ability or social behaviour. They quickly learned the rewarding cues and strongly preferred them in both studies (see electronic supplementary material, figures S1 here and in Aljadeff *et al*. [36]). On the other hand, the change in initial task difficulty was a priori designed to cause the observed change in behaviour, and as we discuss below, its effect is also consistent with the evidence.

There are few possible explanations for how increasing initial task difficulty could have resulted in such a radical change in learning dynamics. One is that in contrast to the previous study, initial task difficulty caused naive sparrows to scrounge more on wells of the demonstrated cue that were opened by the demonstrators. Although many of these wells were emptied, some still contained a few seeds, so that repeated visits to such wells could have possibly encouraged naive sparrows to prefer the demonstrated cue despite lower success rate per visit. Our analysis suggests, however, that this was not the case. Fast learners, and learners that strongly preferred the demonstrated cue, scrounged less or even didn't scrounge at all (see Results and electronic supplementary material, figure S2). It is therefore much more likely that the conformity exhibited by the naive sparrows was based on directly observing the demonstrators and copying their behaviour. Some forms of observational learning have been demonstrated in passerine species (e.g. [37,44–48]), as well as in our house sparrow population [49,50]. Moreover, we have recently shown that our sparrows can copy the same task solution used in the present study (opening wells covers with the demonstrated cue) when paired with a demonstrator in an adjacent cage where no scrounging is possible [39]. Thus, some type of observational learning or imitation seems to be the most likely explanation of our results.

The same observational learning did not result in conformity in the previous study. This could be because the sparrows in the previous study were pre-trained to open unmarked covers; therefore they could start opening covers without paying attention to the demonstrators, and then potentially learn from their own experience that the non-demonstrated rewarding cue is the most rewarding one [36]. In the present study, on the other hand, we omitted the pre-training stage, so that naive sparrows had to learn the action of cover opening. Indeed, a recent study in our laboratory confirmed that sparrows are unlikely to learn to open the covers without gradual shaping or social demonstration [39]. Thus, as hypothesized in the Introduction section, making the task initially more difficult encouraged the naive birds to pay attention to the behaviour of the demonstrators, facilitating social learning. An attentional shift of this kind, which may also involve giving more weight to social information, is consistent with strategies such as ‘copy when uncertain’ [12,14], as well as with the tendency of unsuccessful foragers, or poor learners, to follow others [51,52]. A tendency to rely on social information in such cases, which may generally be adaptive, may also explain why the sparrows continued to prefer the demonstrated cue, which in this particular setup was not adaptive. Thus, our results suggest that sub-optimal conformity may develop locally under some conditions as a result of a strategy that is adaptive on average.

There are two possible non-mutually exclusive interpretations for the learning process observed in our study. The first assumes that sparrows learn food-cue association equally well when they open intact wells and when they visit previously opened wells (i.e. during delayed scrounging). Here, the naive sparrows in our study could have learned the lower success rate when visiting the rapidly depleted demonstrated cue (figure 5) but continued to prefer it because they gave more weight to social information (as suggested above). The second possibility is that sparrows learnt food-cue association only (or mostly) when they open intact wells, and were less attentive to cues when they scrounged on opened wells. In fact, the results of some of our previous studies are consistent with this interpretation [43,49,53] (see also [54]). Importantly, if the sparrows learn food-cue association only when opening intact wells, then, the success rate they experienced with both rewarding cues (demonstrated and alternative) is actually equal. Competition and food depletion will affect the availability of intact wells of the different types, but intact wells of the two rewarding cues always provide seven seeds with a probability of 0.5.

This second interpretation of the learning process is at first puzzling. If learners experience the same success rate with both cues (learning only when opening intact wells), it is not clear why they developed significant opposing preferences in the two studies, rather than exhibiting no preference in both? However, we have recently found that facing two equally rewarding cues (with the same mean and variance), sparrows tend to develop a preference for the one they use more frequently (Flickstein *et al*. unpublished data), a tendency predicted by some learning models [55]. With this type of learning, naive sparrows in the previous study that could quickly explore both types of intact wells (because they already knew to open covers) opened intact wells of the alternative rewarding cue most frequently (because they were not opened by the demonstrators and were therefore more available), which resulted in developing a preference for them. In the present study, on the other hand, the need to socially learn the action of cover opening resulted in initial inclination to open mostly intact wells of the demonstrated cue, and hence in developing a preference towards them.

We believe that, in practice, both interpretations are correct and that the learning process observed in our study involves both effects. It is unlikely that sparrows learned food-cue association only when opening intact wells, especially because the covers of some of the previously opened wells returned to their place (they were somewhat elastic) so they could be perceived by the sparrows as intact wells. Consequently, the perceived success rate of the demonstrated cue would be lower than of the alternative rewarding cue as a result of depletion, favouring the first interpretation of giving higher weight to social information despite lower success rate. On the other hand, given the findings on learning in sparrows that were mentioned above, it is quite possible that sparrows are indeed less attentive to food-related cues during scrounging, and at the same time, develop a preference for the commonly used cue when foraging from intact wells. These combined effects may actually help explain their sub-optimal conformity. First, conformity may be more likely if the difference in success rate that can act against it is perceived as smaller than reflected in our figure 5 (that includes all peck in previously opened wells). Second, adding a tendency to prefer the commonly used option (when both are perceived as equally attractive) can boost the developing preference and explain its persistency.

Further work is needed to examine for how long the sub-optimal conformity exhibited by the sparrows would persist. Although we doubled the length of the experimental stage in comparison to the previous study, the sparrows continued to conform and even increased their tendency to conform (figure 4). Moreover, this was despite the fact that most naive individuals (18 out of the 22) explored the alternative rewarding cue and had sufficient time to learn that it yields a higher success rate (see figures 5 and 6). Yet, we cannot rule out the possibility that extending the experiment even further would allow the naive sparrows to gradually give more weight to asocial information [36,43,49,53]. Alternatively, the sparrows' frequent use of the demonstrated cue, which still yielded some success (especially when opening intact wells) may stabilize sub-optimal conformity as a persistent habit. Interestingly, great tits did not continue to conform after their reward regime was changed to favour the rarely used task solution, giving no indication that they can be trapped in sub-optimal conformity [56]. However, the change applied in this study was based on changing food type, which may be much more salient for the birds than the success rate alone. Clearly, the persistency of sub-optimal conformity in animal societies is an empirical question awaiting further research.

In conclusion, our study shows that manipulating a task in a way that increases learners’ initial dependency on social demonstration can profoundly change the learning dynamics, causing social animals previously shown to exhibit adaptive diversity to develop sub-optimal conformity. These results suggest that variation in task-related cognitive demands may play a much greater role in determining how information is transmitted and shared among group members than previously appreciated. Our study highlights the need for greater attention into the role of task-related factors on the reliance of social learning, use of social learning strategies, and emergence of group-level culture.

## Data Availability

The data supporting the findings of this study are available in a supplementary file [57] and on Dryad Digital Repository: https://doi.org/10.5061/dryad.9w0vt4bkt [58].

## References

[1] Galef BG, Giraldeau L-A. 2001 Social influences on foraging in vertebrates: causal mechanisms and adaptive functions. Anim. Behav. **61**, 3-15. (10.1006/anbe.2000.1557) 11170692

[2] Reader SM, Laland KN. 2003 Animal innovation. Oxford, UK: Oxford University Press.

[3] Allen J, Weinrich M, Hoppitt W, Rendell L. 2013 Network-based diffusion analysis reveals cultural transmission of lobtail feeding in humpback whales. Science **340**, 485-488. (10.1126/science.1231976) 23620054

[4] Whiten A. 2017 Culture extends the scope of evolutionary biology in the great apes. Proc. Natl Acad. Sci. USA **114**, 7790-7797. (10.1073/pnas.1620733114) 28739927 PMC5544264

[5] Danchin E, Giraldeau L-A, Valone TJ, Wagner RH. 2004 Public information: from nosy neighbors to cultural evolution. Science **305**, 487-491. (10.1126/science.1098254) 15273386

[6] Creanza N, Kolodny O, Feldman MW. 2017 Cultural evolutionary theory: How culture evolves and why it matters. Proc. Natl Acad. Sci. USA **114**, 7782-7789. (10.1073/pnas.1620732114) 28739941 PMC5544263

[7] Whiten A, Mesoudi A. 2008 Establishing an experimental science of culture: animal social diffusion experiments. Phil. Trans. R. Soc. B **363**, 3477-3488. 18799418 10.1098/rstb.2008.0134 PMC2607342

[8] Heyes CM. 1994 Social learning in animals: categories and mechanisms. Biol. Rev. **69**, 207-231. 8054445 10.1111/j.1469-185x.1994.tb01506.x

[9] Hoppitt W, Laland KN. 2013 Social learning: An introduction to mechanisms, methods, and models. Princeton, NJ: Princeton University Press.

[10] Fragaszy DM, Perry S. 2003 The biology of traditions. Cambridge, UK: Cambridge University Press.

[11] Laland KN, Hoppitt W. 2003 Do animals have culture? Evol. Anthropol. **12**, 150-159. (10.1002/evan.10111)

[12] Laland KN. 2004 Social learning strategies. Anim. Learn. Behav. **32**, 4-14. (10.3758/BF03196002) 15161136

[13] Kendal RL, Boogert NJ, Rendell L, Laland KN, Webster M, Jones PL. 2018 Social learning strategies: bridge-building between fields. Trends Cogn. Sci. **22**, 651-665. (10.1016/j.tics.2018.04.003) 29759889

[14] Smolla M, Alem S, Chittka L, Shultz S. 2016 Copy-when-uncertain: bumblebees rely on social information when rewards are highly variable. Biol. Lett. **12**, 208-213. (10.1098/rsbl.2016.0188) PMC4938046 27303053

[15] Toelch U, Bruce MJ, Newson L, Richerson PJ, Reader SM. 2014 Individual consistency and flexibility in human social information use. Proc. Biol. Sci. **281**, 20132864. (10.1098/rspb.2013.2864) 24352950 PMC3871325

[16] Heyes C, Pearce JM. 2015 Not-so-social learning strategies.

[17] Aisner R, Terkel J. 1992 Ontogeny of pine cone opening behaviour in the black rat, Rattus rattus. Anim. Behav. **44**, 327-336. (10.1016/0003-3472(92)90038-B)

[18] Farine DR, Spencer KA, Boogert NJ. 2015 Early-life stress triggers juvenile zebra finches to switch social learning strategies. Curr. Biol. **25**, 2184-2188. (10.1016/j.cub.2015.06.071) 26212879 PMC4540255

[19] Creanza N, Fogarty L, Feldman MW. 2012 Models of cultural niche construction with selection and assortative mating. PLoS One **7**, e42744. 22905167 10.1371/journal.pone.0042744 PMC3419226

[20] Katsnelson E, Lotem A, Feldman MW. 2014 Assortative social learning and its implications for human and animal societies. Evolution (N. Y) **68**, 1894-1906. (10.1111/evo.12403) 24628026

[21] Henrich J, Boyd R. 1998 The evolution of conformist transmission and the emergence of between-group differences. Evol. Hum. Behav. **19**, 215-241. (10.1016/S1090-5138(98)00018-X)

[22] Boyd R, Richerson PJ. 2005 The origin and evolution of cultures. Oxford, UK: Oxford University Press.

[23] Morgan TJH, Laland KN. 2012 The biological bases of conformity. Front. Neurosci. **6**, 1-7. (10.3389/fnins.2012.00087) 22712006 PMC3375089

[24] Claidière N, Whiten A. 2012 Integrating the study of conformity and culture in humans and nonhuman animals. Psychol. Bull. **138**, 126-145. 22061691 10.1037/a0025868

[25] Whiten A, Horner V, De Waal FBM. 2005 Conformity to cultural norms of tool use in chimpanzees. Nature **437**, 737-740. (10.1038/nature04047) 16113685

[26] Van De Waal E, Borgeaud C, Whiten A. 2013 Potent social learning and conformity shape a wild primate's foraging decisions. Science **340**, 483-485. (10.1126/science.1232769) 23620053

[27] Pike TW, Laland KN. 2010 Conformist learning in nine-spined sticklebacks' foraging decisions. Biol. Lett. **6**, 466-468. (10.1098/rsbl.2009.1014) 20129948 PMC2936200

[28] Aplin LM, Farine DR, Morand-Ferron J, Cockburn A, Thornton A, Sheldon BC. 2015 Experimentally induced innovations lead to persistent culture via conformity in wild birds. Nature **518**, 538-541. (10.1038/nature13998) 25470065 PMC4344839

[29] Whiten A. 2019 Cultural evolution in animals. Annu. Rev. Ecol. Evol. Syst. **50**, 27-48.

[30] Nöbel S, Jacquet A, Isabel G, Pocheville A, Seabright P, Danchin E. 2022 Conformity in mate choice, the overlooked social component of animal and human culture. Biol. Rev. **98**, 132-149. (10.1111/brv.12899) 36173001 PMC10087591

[31] Leeuwen EV, Kendal RL, Tennie C, Haun DBM. 2015 Conformity and its look-a-likes. Anim. Behav. **110**, e1-e4. (10.1016/j.anbehav.2015.07.030)

[32] Aplin LM, Farine DR, Morand-ferron J, Cockburn A, Thornton A, Sheldon BC. 2015 Counting conformity : evaluating the units of information in frequency-dependent social learning. Anim. Behav. **110**, e5-e8. (10.1016/j.anbehav.2015.09.015)

[33] Smaldino PE, Aplin LM, Farine DR. 2018 Sigmoidal Acquisition Curves Are Good Indicators of Conformist Transmission. Sci. Rep. **8**, 1-10. (10.1038/s41598-018-30248-5) 30228351 PMC6143626

[34] Giraldeau L. 1984 Group foraging: the skill pool effect and frequency-dependent learning. Am. Nat. **124**, 72-79.

[35] Smolla M, Gilman RT, Galla T, Shultz S. 2015 Competition for resources can explain patterns of social and individual learning in nature. Proc. R. Soc. B **282**, 20151405. (10.1098/rspb.2015.1405) PMC4614750 26354936

[36] Aljadeff N, Giraldeau LA, Lotem A. 2020 Competitive advantage of rare behaviours induces adaptive diversity rather than social conformity in skill learning: ADAPTIVE DIVERSITY in SKILL LEARNING. Proc. R. Soc. B **287**, 1-6. (10.1098/rspb.2020.1259/rspb20201259) PMC7482281 32811312

[37] Aplin LM, Sheldon BC, Morand-Ferron J. 2013 Milk bottles revisited: social learning and individual variation in the blue tit, Cyanistes caeruleus. Anim. Behav. **85**, 1225-1232. (10.1016/j.anbehav.2013.03.009)

[38] Garcia-Nisa I, Evans C, Kendal RL. 2023 The influence of task difficulty, social tolerance and model success on social learning in Barbary macaques. Sci. Rep. **13**, 1176. 36670123 10.1038/s41598-022-26699-6 PMC9860066

[39] Zurek N. 2021 Learning successfully from others: the effect of different types of social demonstration on solving different components of a foraging task (Unpublished Master's Thesis). Tel Aviv: Tel-Aviv University.

[40] Aljadeff N, Lotem A. 2021 Task-dependent reversal learning dynamics challenge the reversal paradigm of measuring cognitive flexibility. Anim. Behav. **179**, 183-197. (10.1016/j.anbehav.2021.07.002)

[41] Dor R, Lotem A. 2009 Heritability of nestling begging intensity in the house sparrow (Passer domesticus). Evolution (N. Y) **63**, 738-748. (10.1111/j.1558-5646.2008.00598.x) 19210530

[42] Katsnelson E, Motro U, Feldman MW, Lotem A. 2008 Early experience affects producer-scrounger foraging tendencies in the house sparrow. Anim. Behav. **75**, 1465-1472. (10.1016/j.anbehav.2007.09.020)

[43] Ilan T, Katsnelson E, Motro U, Feldman MW, Lotem A. 2013 The role of beginner's luck in learning to prefer risky patches by socially foraging house sparrows. Behav. Ecol. **24**, 1398-1406. (10.1093/beheco/art079) 24137046 PMC3796710

[44] Brodin A, Utku Urhan A. 2014 Sex differences in learning ability in a common songbird, the great tit—females are better observational learners than males. Behav. Ecol. Sociobiol. **69**, 237-241. (10.1007/s00265-014-1836-2)

[45] Emery NJ, Clayton NS. 2001 Effects of experience and social context on prospective caching strategies by scrub jays. Nature **414**, 443-446. (10.1038/35106560) 11719804

[46] Fritz J, Kotrschal K. 1999 Social learning in common ravens, Corvus corax. Anim. Behav. **57**, 785-793. (10.1006/anbe.1998.1035) 10202087

[47] Riebel K, Spierings MJ, Holveck M-J, Verhulst S. 2012 Phenotypic plasticity of avian social-learning strategies. Anim. Behav. **84**, 1533-1539. (10.1016/j.anbehav.2012.09.029)

[48] Hoppitt W, Laland KN. 2008 Chapter 3 Social Processes Influencing Learning in Animals: A Review of the Evidence. Adv. Study Behav. **38**, 105-165. (10.1016/S0065-3454(08)00003-X)

[49] Truskanov N, Lotem A. 2015 The importance of active search for effective social learning: An experimental test in young passerines. Anim. Behav. **108**, 165-173. (10.1016/j.anbehav.2015.07.031)

[50] Truskanov N, Lotem A. 2017 Trial-and-error copying of demonstrated actions reveals how fledglings learn to ‘imitate’ their mothers.

[51] Barta Z, Giraldeau LA. 1998 The effect of dominance hierarchy on the use of alternative foraging tactics: A phenotype-limited producing-scrounging game. Behav. Ecol. Sociobiol. **42**, 217-223. (10.1007/s002650050433)

[52] Katsnelson E, Motro U, Feldman MW, Lotem A. 2011 Individual-learning ability predicts social-foraging strategy in house sparrows. Proc. Biol. Sci. **278**, 582-589. (10.1098/rspb.2010.1151) 20810440 PMC3025675

[53] Truskanov N, Shy R, Lotem A. 2018 Context-specific learning and its implications for social learning. Behav. Ecol. **29**, 1046-1055. (10.1093/beheco/ary078)

[54] Giraldeau L, Lefebvre L. 1987 Scrounging prevents cultural transmission of food-finding behaviour in pigeons. Anim. Behav. **35**, 387-394.

[55] Roth A, Erev I. 1995 Learning in extensive-form games: Experimental data and simple dynamic models in the intermediate term. Games Econ. Behav. **8**, 164-212.

[56] Aplin LM, Sheldon BC, McElreath R. 2017 Conformity does not perpetuate suboptimal traditions in a wild population of songbirds. Proc. Natl Acad. Sci. USA **114**, 7830-7837. (10.1073/pnas.1621067114) 28739943 PMC5544276

[57] Marković Đ, Aljadeff N, Aplin LM, Lotem A. 2023 Increased initial task difficulty drives social foragers to develop sub-optimal conformity instead of adaptive diversity. *Figshare*. (10.6084/m9.figshare.c.6725578) PMC10320340 37416826

[58] Marković Đ, Aljadeff N, Aplin LM, Lotem A. 2023 Increased initial task difficulty drives social foragers to develop sub-optimal conformity instead of adaptive diversity. *Dryad Digital Repository*. (10.5061/dryad.9w0vt4bkt) PMC10320340 37416826

